# Small-Molecule Inhibitor Targeting Protein Kinase D: A Potential Therapeutic Strategy

**DOI:** 10.3389/fonc.2021.680221

**Published:** 2021-06-24

**Authors:** Die Lv, Hongli Chen, Yun Feng, Bomiao Cui, Yingzhu Kang, Ping Zhang, Min Luo, Jiao Chen

**Affiliations:** State Key Laboratory of Oral Diseases, National Clinical Research Center for Oral Diseases, West China Hospital of Stomatology, Sichuan University, Chengdu, China

**Keywords:** protein kinase D, small-molecule inhibitor, function, therapeutics, diseases

## Abstract

The protein kinase D (PKD) family is a family of serine-threonine kinases that are members of the calcium/calmodulin-dependent kinase (CaMK) superfamily. PKDs have been increasingly implicated in multiple pivotal cellular processes and pathological conditions. PKD dysregulation is associated with several diseases, including cancer, inflammation, and obesity. Over the past few years, small-molecule inhibitors have emerged as alternative targeted therapy with fewer adverse side effects than currently available chemotherapy, and these specifically targeted inhibitors limit non-specific toxicities. The successful development of PKD inhibitors would significantly suppress the growth and proliferation of various cancers and inhibit the progression of other diseases. Various PKD inhibitors have been studied in the preclinical setting. In this context, we summarize the PKD inhibitors under investigation and their application for different kinds of diseases.

## Introduction

The protein kinase D (PKD) family is a serine-threonine kinase family that is a subfamily of the calcium/calmodulin-dependent kinase (CaMK) superfamily. There are three isoforms (PKD1, PKD2, and PKD3) that share high sequence identity in mammals. In 1994, PKD1 (first termed PKCμ) was characterized as a novel kinase activated by the secondary messenger diacylglycerol (DAG) (1, 2). PKD3 and PKD2 were subsequently discovered in 1999 (3) and 2001 (4), respectively. The PKDs are activated by many substances, including phorbol ester, platelet-derived growth factor, and G protein-coupled receptor ligands (5). In humans, the three PKD isoforms are expressed in most tissues, including the oral cavity, skin, liver, lung, pancreas, breast, and stomach (4, 6–10). Interestingly, the expression of different isoforms may be different depending on the physiological and pathological conditions. For example, PKD1 expression is high in normal breast tissue, while PKD2 and PKD3 expression levels are low. In contrast, PKD1 expression is absent and PKD2 and PKD3 expression levels are elevated in breast cancer (11). In animals, PKD1, PKD2, and PKD3 are widely expressed in the central nervous system, large intestine, adrenal gland, bladder, cerebellum, and colon, and the expression levels of the three isoforms are different (12–15). PKD activity is associated with many diseases and pathological conditions, such as cancer, metabolic diseases, and inflammation, where it regulates cell proliferation, differentiation, programmed cell death, migration, and invasion (16–19). Pharmacological targeting of PKDs to regulate their expression and kinase activity has important implication for treating disease (20).

Small-molecule inhibitors (SMIs) have been accepted as new therapeutic alternatives for treating disease because they can provide a targeted treatment approach, resulting in fewer undesirable side effects than currently available chemotherapy (21). SMIs are compounds of less than 500 Da, that can potentially bind to a wide range of extracellular and intracellular targets. PKDs have attracted the interest of many researchers because they can regulate cellular functions and play important roles in disease; however, there are no clinical inhibitors targeting PKDs (20). Recently, specific PKD inhibitors have been discovered, and confirmed the important role of the PKDs in the induction and progression of many diseases (22–24).

Over the past few decades, studies have shown that PKDs play an important role in the occurrence and development of various diseases (25). Many PKD SMIs have been investigated in preclinical studies as unique and effective potential drugs. In this review, we summarize the known PKD SMIs and their potential application in various diseases.

## PKD Structure and Function

**Figure 1:**
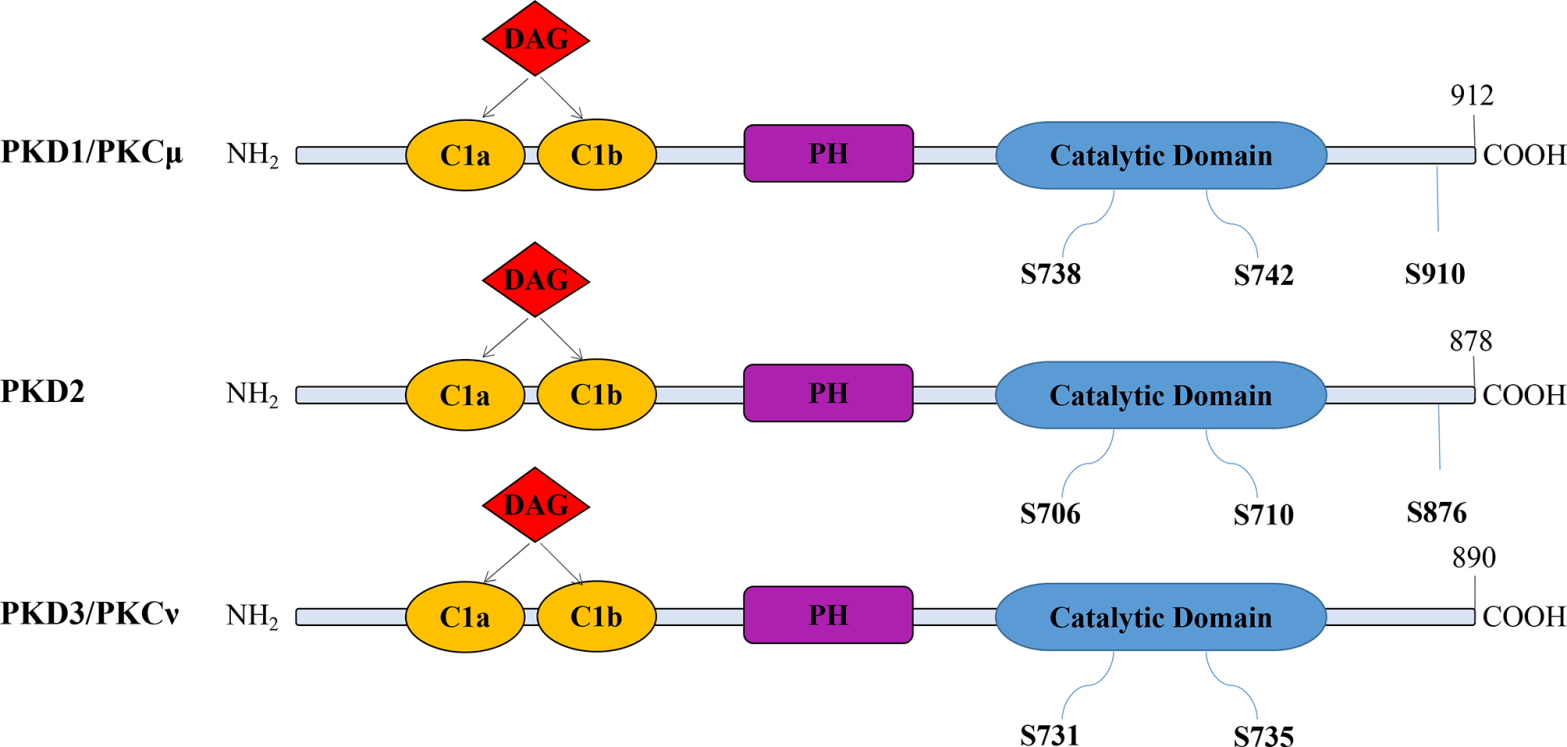
The modular structure of PKD isoforms. The PKD isoforms have highly homologous structures containing cysteine-rich Zn-finger-like motifs (CIa and CIb), a pleckstrin homology (PH) domain, and a C-terminal catalytic domain. PKDs are activated by the phosphorylation of serine residues. However, PKD3 lacks a C-terminal autophosphorylation site within the PDZ (PSD-95/Discs large/ZO-1) binding motif present in both PKD1 and PKD2 (S910 in PKD1; S876 in PKD2).

The PKD family members have a similar structure but different activation patterns and downstream functions (26). They contain a catalytic domain, a pleckstrin homology (PH) domain, and two N-terminal cysteine-rich zinc finger domains (C1a and C1b), which bind DAG and phorbol esters (27). The three isoforms have high homology, especially in the catalytic and C1 domains. Their differences are located in the N-terminal region and the regions flanked by the C1 and PH domains, possibly related to their functional differences (28). In the absence of the PH structure, enzyme activity increases, indicating that the PH domain inhibits enzyme activity (29) (**Figure 1**).

PKD dysfunction is associated with many pathological processes, including promoting cell proliferation, inhibiting apoptosis, promoting angiogenesis, and disrupting homeostasis (4, 20). Different isoforms can promote tumorigenesis and anti-tumor effects (25). In cancer, PKD1 plays different roles depending on tumor type, both pro-tumorigenesis and anti-tumor. PKD1 levels are significantly downregulated in breast cancer, acting as an antioncogene (11). In pancreatic cancer, PKD1 levels are upregulated. These increased levels promote mitosis and angiogenesis by mediating signal transduction and enhance chemical resistance and invasion of pancreatic cancer cells (30). Compared to PKD1, PKD2 and PKD3 are often associated with tumor progression. Hao et al. (31) have reported that PKD2 mediates endothelial cell proliferation, migration, and angiogenesis. In addition, PKD2 is involved in the development of various tumor types including pancreatic, colorectal, and breast cancer, glioma multiforme, and leukemia. It plays a versatile role in these cancers through uncontrolled growth, survival, neovascularization, metastasis, and invasion (32). The study showed that PKD3 enhances proliferation of cancer cells through the mTORC1-S6K1 signaling pathway in triple negative breast cancer (33). Silencing PKD3 also inhibited tumor immune escape by reducing PD-L1 expression in oral squamous cell carcinoma cells (OSCC) (7). Thus, PKDs play important roles in disease and are potential targets for treatment.

## PKD Inhibitors

SMIs are typically chemically synthesized organic compounds with molecular weights of less than 500 Da. Over the past few decades, chemotherapy has been a key treatment for fighting cancer; however, it usually does not distinguish between normal and tumor cells and is limited by a narrow treatment index. In contrast, targeted therapies interfere with specific molecular targets, limiting non-specific toxicity. Compared with monoclonal antibody drugs that only act on cell surface receptors, small molecular inhibitors can enter the cell *via* a concentration gradient so that they have a broader range of targets (21). Small-molecule inhibitors have been investigated in cancer and cardiovascular, endocrine, infectious, immune, metabolic, and nervous system diseases (25). More and more PKD inhibitors have been identified, and pan-PKD inhibitors have demonstrated *in vitro* and *in vivo* antitumoral activity in various disease models, despite the opposing roles of the PKD isoforms in some disease (34, 35).

### CRT0066101

**Figure 2:**
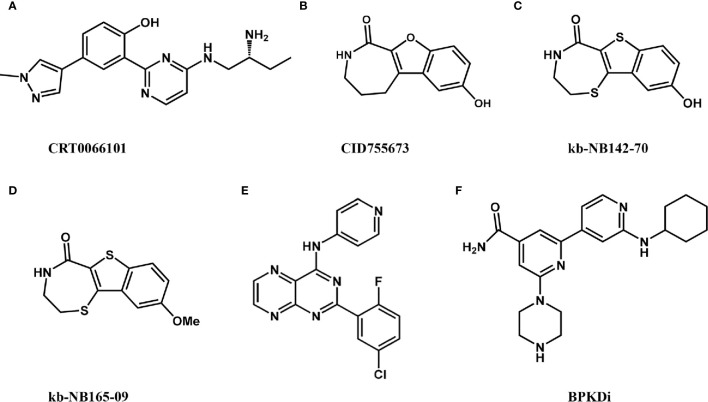
Reported PKD inhibitors.

In 2010, Harikumar et al. (23) discovered CRT0066101 (2-[4-((2R)-2-aminobutyl-amino)-pyrimidin-2-yl]-4-(1-methyl-1H-pyrazol-4-yl)-phenol dihydrochloride) (**Figure 2A**), which is the most effective, selective, orally bioavailable pan-PKD inhibitor (IC_50_ = 1, 2.5, and 2 nM for PKD1, 2, and 3, respectively). CRT0066101 can inhibit tumor growth in animal models of breast, colorectal, and pancreatic cancer (23, 36, 37). In addition, CRT0066101 exerts synergistic effects against colorectal cancer with regorafenib (38), an FDA-approved multi-kinase inhibitor for the treatment of metastatic colorectal cancer. The anti-tumor effects of CRT0066101 are mediated its inhibition of cell growth, and angiogenesis and its promotion of apoptosis. Another study showed that CRT0066101 could inhibit virus replication (39). These studies showed that the compound is well tolerated in mice with no significant side effects, making CRT0066101 an ideal candidate for clinical development.

### CID755673

CID755673 (2,3,4,5-Tetrahydro-7-hydroxy-1H-benzofuro[2,3-c] azepin-1-one) was, identified by high-throughput screening, and is a powerful, selective, cell-active PKD inhibitor (**Figure 2B**). Interestingly, CID755673 inhibits PKD1 with an IC_50_ of 182 nM and demonstrated selective PKD1 inhibition compared to three different PKC isoforms, AKT, polo-like kinase 1 (PLK1), CDK-activating kinase (CAK), and CAMKII. This compound does not compete with ATP for enzyme inhibition. CID755673 can inhibit cell proliferation and metastasis, phorbol ester-induced nuclear exclusion of class IIa histone deacetylase 5 (HDAC5), and transport of protein from the Golgi to the plasma membrane (22). Furthermore, CID755673 inhibits the pro-angiogenic effect of astragaloside IV by blocking the PKD1-HDAC5-VEGF signaling pathway, to some extent (40). CID755673 can maintain the undifferentiated state of mouse embryonic stem cells (ESCs) when combined with a mitogen-activated protein kinase kinase (MEK) inhibitor by increasing AKT phosphorylation and activating PI3K/AKT signaling (12).

### kb-NB142-70 and kb-NB165-09

kb-NB142-70 (**Figure 2C**) and its methoxy-analog kb-NB165-09 (**Figure 2D**) are structural analogs of CID755673 with two- to six-fold improved selectivity over CID755637. In particular, modification of the aromatic core significantly increased potency while maintaining high PKD specificity. Both kb-NB142-70 and kb-NB165-09 inhibit the proliferation of prostate cancer cells by inducing G2/M cell cycle arrest. Importantly, these analogs dramatically arrest cell proliferation, increase cytotoxicity, and inhibit cell migration and invasion (41). Although toxicity to tumor cells and inhibition of cell proliferation by these analogs are significantly increased, they are rapidly degraded *in vivo*, reducing their anti-tumor effects (41). Therefore, further optimization of these analogs required to increase their metabolic stability.

### SD-208

The pteridine analog SD-208 is an ATP-competitive pan-PKD SMI (**Figure 2E**), with IC_50_s of 107, 94, and 105 nM for PKD1, PKD2, and PKD3, respectively. The first PKD-related SAR (structure-activity relationship) of this structure was established by a series of SD-208 analogs and demonstrated that an electron-deficient aromatic ring is preferred in zone 1 and 4-aminopyridine in zone 2 is essential for activity. SD-208 exhibited a narrow SAR profile with low nanomolar potency. SD-208 is bioactive *in vitro* and *in vivo* and can effectively inhibit cells proliferation, and reduce cell invasion and survival of cancer cells (24). However, SD-208 has also been reported as a transforming growth factor βreceptor I (TGF-Bri) inhibitor that blocks tumor invasion and metastasis (42, 43). Therefore, its anti-tumor effects may be mediated in part by its inhibition of (TGF-βRI) (42).

### BPKDi

Bipyridyl PKD inhibitor (BPKDi) is a selective PKDi identified through high throughput screening and pharmaceutical chemistry (**Figure 2F**) (44, 45). BPKDi inhibits all three isoforms of the PKD family, with IC_50_s of 1, 9, and 1 nM, respectively. PKD promotes the phosphorylation of class IIA HDACs, which induces pathological cardiac hypertrophy (44, 46). BPKDi can abolish the phosphorylation and nuclear export of the class IIa HDACs in cardiomyocytes, concomitantly suppressing hypertrophy of these cells (44).

### Other Inhibitors

PKD inhibitors of other chemotypes have been profiled in disease areas beyond cancer. Examples include the 3,5-diarylisoxazoles and 2,6-naphthyridines that potently inhibit PKD and are being investigated as potential treatments for cardiac hypertrophy (47, 48). The 3, 5-diarylisoxazoles are not only active in cell assays of phosphorylation-dependent HDAC5 nuclear export but are, also orally bioavailable, and highly selective compared to a panel of additional putative histone deacetylase (HDAC) kinases (47). The 2,6-naphthyridines have similar effects to the 3,5-diarylisoxazoles, but the PKD selectivity is not very high. Indeed, it also can inhibit PKC to some degree (48). Both 1-NA-PP1 and IKK-16 were identified as pan-PKD inhibitors through a small-scale targeted kinase inhibitor library screen. They are ATP-competitive inhibitors that have high potency and selectivity for PKD. SAR analysis suggests that 1-NA-PP1 is considerably more potent than IKK-16, with distinct substituent effects at the pyrazolopyrimidine core (49). Another compound (3-IN-PP1) is 10-fold more potent than 1-NM-PP1 (the lead compound of 3-IN-PP1), with strong anti-proliferation activity against PANC-1 cells (50, 51).

## PKD Inhibitors as Effective Therapeutic Strategies Against Disease

### Cancer

Pancreatic ductal adenocarcinoma (PDAC) accounts for 90% of all pancreatic cancers and is considered one of the deadliest cancers with a 5-year survival rate of about 3–5% (52). Many studies have shown that PKD is involved in various pathological processes in pancreatic cancer, including cell proliferation, invasion, energy metabolism, and drug resistance (30, 35, 53). CRT0066101 can inhibit cell proliferation, induce apoptosis, reduce PKD1/2 activity induced by neurotensin, reduce neurotensin-induced PKD-mediated Hsp27 phosphorylation, inhibit NF- κB activation, and block NF-κB-dependent proliferation and survival in pancreatic cancer. CRT0066101 also inhibits the proliferation and growth of human PANC-1 PDAC xenografts, indicating that PKD is a potential therapeutic target for this cancer (23).

Moreover, early pancreatic lesions characterized by ADM, the transdifferentiation of pancreatic acinar cells to a ductal phenotype, are important inducers of pancreatic cancer. AMD may be transformed into pancreatic intraepithelial lesions (PanINs) in the progress of activating Kras mutations or persistent epidermal growth factor receptor (EGF-R) signaling, eventually leading to the occurrence of pancreatic cancer. Liou et al. (54) identified PKD1 as an essential signaling protein downstream of Kras and EGF-R that can drive the formation of such duct-like cells. Interestingly, this process can be reversed by PKD silencing or with PKD inhibitors kb-NB-142-70 and CRT0066101. These studies demonstrated that PKD is an effective drug target, and PKD-specific inhibitors may have important role in the treatment and prognosis of pancreatic cancer patients.

Several studies have shown that various PKD SMIs can inhibit pathological processes in prostate cancer cells. In particular, the PKD inhibitor (CID755673) and its analogs inhibit the proliferation, migration, and invasion of prostate cancer cells by blocking cell cycle progression at G2/M (24, 41). In addition, Chen et al. (55) have suggested that CID755673 and KB-NB142-70 can enhance PMA-induced PARP cleavage in prostate cancer cells by inhibiting PKD activity, thus synergizing with PMA to promote apoptosis.

Unlike PKD1 and PKD2, PKD3 expression is highly upregulated in ER negative breast cancer. Normally, the estrogen receptor (ER) directly binds to the PRKD3 gene promoter to inhibit PKD3 expression. Loss of the ER leads to PKD3 upregulation, resulting in all the hallmarks of aggressive invasive ductal carcinomas (IDC), including tumor progression (36). Targeting PKD in ER-negative breast cancer with the selective PKD inhibitor CRT0066101 or PKD3 knockdown causes decreased growth and metastasis of primary tumors *in vivo*. These data support the development of PKD inhibitors for clinical use against ER-negative breast cancer (36). Furthermore, PRKD2 and PRKD3 are preferentially expressed in triple-negative breast cancer (TNBC) (56). CRT0066101 inhibits the proliferation of TNBC cells and increases apoptosis and the G1-phase population *in vitro*. Similarly, this inhibitor reduces breast tumor volume *in vivo*. These anti-TNBC effects by CRT0066101 are mediated by inhibiting the phosphorylation of MYC, MAPK1/3, AKT, and YAP (56).

PKD-specific inhibitors are also being investigated in various other cancers. For instance, CRTO066101 can cause a dose-dependent suppression of PKD2 activation in colorectal cancer (CRC) cells, resulting in G2/M arrest and apoptosis. The researchers further found a series of signaling events affected by CRT0066101, including PARP cleavage, caspase-3 activation, AKT and ERK signaling suppression, and increased NF-κB activity. Moreover, daily administration of CRT0066101 significantly inhibits the growth of HCT116 xenograft in nude mice. Taken together, these data suggest that PKD plays an important role in regulating growth signals in CRC, and its inhibition by small molecular inhibitors may represent a new treatment for this cancer (37). Additionally, CRT0066101 can suppress bladder cancer growth by inhibiting PKD2 and blocking cell cycle progression at G2/M (57).

All in all, PKD is a likely target in multiple cancers, and PKD SMIs are showing promising results as cancer chemotherapy.

### Inflammation

The inflammatory response plays a pivotal role in acute pancreatitis, and studies have shown that PKD is involved in regulating inflammation. Inhibition of PKD by the specific PKD inhibitor CRT0066101 alleviates the symptoms of pancreatitis (58, 59). Specifically, the inhibitors attenuate the inflammatory response and reduce the severity of the disease by inhibiting NF-κB activation, which decreases inflammatory cell infiltration, pancreatic interleukin-6 (IL-6), and monocyte chemoattractant protein-1 (MCP-1) (58). In addition, inflammatory lysophosphatidic acid (LPA) levels increase microglia migration and promote the pro-inflammatory phenotype through the LPAR5/PKD axis (60). When this axis is inhibited by PKD inhibitor CRT0066101, glial cell migration decreases, cytotoxicity is detected, and the expression and secretion of pro-inflammatory mediators are abrogated (60).

Group B streptococcus (GBS) can infect fetal membranes and cause chorioamnionitis during pregnancy, resulting in adverse pregnancy outcomes. This infection is characterized by the release of inflammatory cytokines, the assembly of NLRP3 inflammatory bodies, and the activation of NF-κB. Interestingly, CRT0066101 can inhibit these responses, suggesting that PKD is involved in mediating the immune response of human placental macrophages to GBS (61). Thus, CRT0066101 may represent an important treatment for chorioamnionitis.

### Obesity

Obesity is one of the strongest predictors of peripheral insulin resistance (62). AMPK (AMP-activated protein kinase, AMPK) is an energy-sensing enzyme that is inhibited by insulin resistance. PKD is emerging as an important regulator of cellular adaptation to an obese environment (63–65). The specific PKD inhibitor CRT0066101 or PKD1 knockdown can eliminate the inhibitory effect on AMPK, illustrating that PKD1 inhibitor restores AMPK signaling to abolish insulin resistance in muscle cells (66). PKD inhibitor CID755673 can also enhance cardiac function in obese and diabetic patients independent of glucose homeostasis, insulin action, and changes in body composition by inhibiting PKD phosphorylation and expression (67). Taken together, these data suggest that PKD inhibitors can abrogate various complications caused by obesity.

### Pathological Hypertrophy

PKD can promote the phosphorylation-dependent nuclear export of HDACs, leading to pathological hypertrophy (46). Therefore, the inhibition of PKD may represent a potential treatment for this disease. Indeed, various specific PKD inhibitors (e.g., BPKDi,3,5-diarylazoles and 2,6-naphthyridine) have been shown to have good efficacy against pathological cardiac hypertrophy, with good bioavailability. PKD inhibition mediated these therapeutic effects by blocking the phosphorylation and nuclear export of HDACs (44, 47, 48).

### Metabolic Bone Diseases

Bones serve as scaffolds for the human body. In bone metabolism, osteoclasts and osteoblasts complement each other. When this balance is broken, or bone cell disorders arise, bone tissue destruction occurs, clinically manifesting as bone tuberculosis and malignant bone tumors. Several studies have shown that the PKD family is important for bone metabolism and pathology. Balanced osteoclast and osteoblast activity is necessary for bone health. PKD plays an important role in osteoclast formation. Treatment of bone marrow macrophages with CRT0066101 or CID755673 prevents them from becoming osteoclasts, indicating the importance of PKD in bone health (68). In another study, CRT6600101 carried by nanomaterials reduced IL-1β-induced inflammatory stress in chondrocytes *via* the NF-κB pathway (69). Overall, PKD inhibitors are a potential treatment for diseases of bone metabolism.

## Conclusion and Perspectives

There is increasing evidence that PKD is a vital signaling molecule, and its dysregulation causes various pathological conditions, including cancer, inflammation, and obesity (**Table 1**). However, the same PKD isoform can have similar or opposite effects in different cancers, and different PKD isoforms can behave differently in the same cancer. In general, PKD can have both anti-cancer and pro-cancer effects in different cells. New evidence indicates that PKD1 acts as a tumor suppressor in breast cancer but is a tumor driver in other cancers (e.g., pancreatic and prostate cancer). Through different mechanisms, PKD1 positively regulates the ERK/MAPK pathway, increases DNA replication, and inhibits apoptosis (70). PKD2 is a pro-tumor protein in various cancers. It activates NF-κB signaling, induces angiogenesis, inhibits apoptosis, and promotes the development of prostate, breast, pancreas, and stomach cancer (32). PRKD3 promotes the proliferation, growth, migration, and invasion of cancer cells in various tumor types (e.g., colorectal, gastric, liver, prostate, and breast cancer), and accumulating evidence suggests that PRKD3 is a promising therapeutic target for cancer treatment (10).

**Table 1:** Inhibition of PKD by various small molecular inhibitors.

Name	Diseases	Cells/Tissues	Signal pathways	References
CRT0066101	Pancreatic cancer	Panc-1, Panc-28, Primary pancreatic acinar cells	NF-κB, HSP27, Notch	(23, 54)
	Breast cancer	MDA-MB-231, MCF-7, T47D, MDA-MB-468	Hormone receptors α(ER-α), MYC, MAPK1/3, AKT, YAP	(36, 56)
	Colorectal cancer	HCT116	AKT, ERK, NF-κB	(37)
	Bladder cancer	TCCSUP, UMUC1, T24T, T24	Cdc25C, CyclinB_1_-CDK1	(57)
	Pancreatitis	Primary pancreatic acinar cells, AR42J	NF-κB	(58, 59)
	Neuroinflammatory	BV-2, Primary microglia	MAPK, AKT, NF-κB, c-Jun, Stat1/3	(60)
	Chorioamnionitis	Primary placental macrophages	NLRP3, NF-κB	(61)
	Obesity	C2C12	AMPK	(66)
	Osteoclasts	Primary osteoclasts	None	(68)
	Chondrocytes	Primary chondrocytes	NF-κB	(69)
CID755673	Prostate cancer,	LNCaP	ERK, NF-κB	(22, 55)
	Diabetic	Heart and right tibia tissue	HADC5	(67)
	Osteoclasts	Primary osteoclasts	None	(68)
SD-208	Prostate cancer	PC3, DU145	Cip1/p21, Cdc25C/Cdc2	(24)
kb-NB142-70,	Prostate cancer	PC-3,	PARP, ERK, NF-κB	(41)
kb-NB165-09	Pancreatic cancer	CFPAC-1, Panc-1		
BPKDi	Cardiac hypertrophy	Primary cardiac myocytes	HDAC5	(50)
3,5-diarylisoxazoles, 2,6-naphthyridines	Cardiac hypertrophy	Primary cardiac myocytes	HDAC5	(47, 48)
1-NA-PP1, 3-IN-PP1	Prostate cancer	PC3, HEK293T	None	(49, 50)

**Figure 3:**
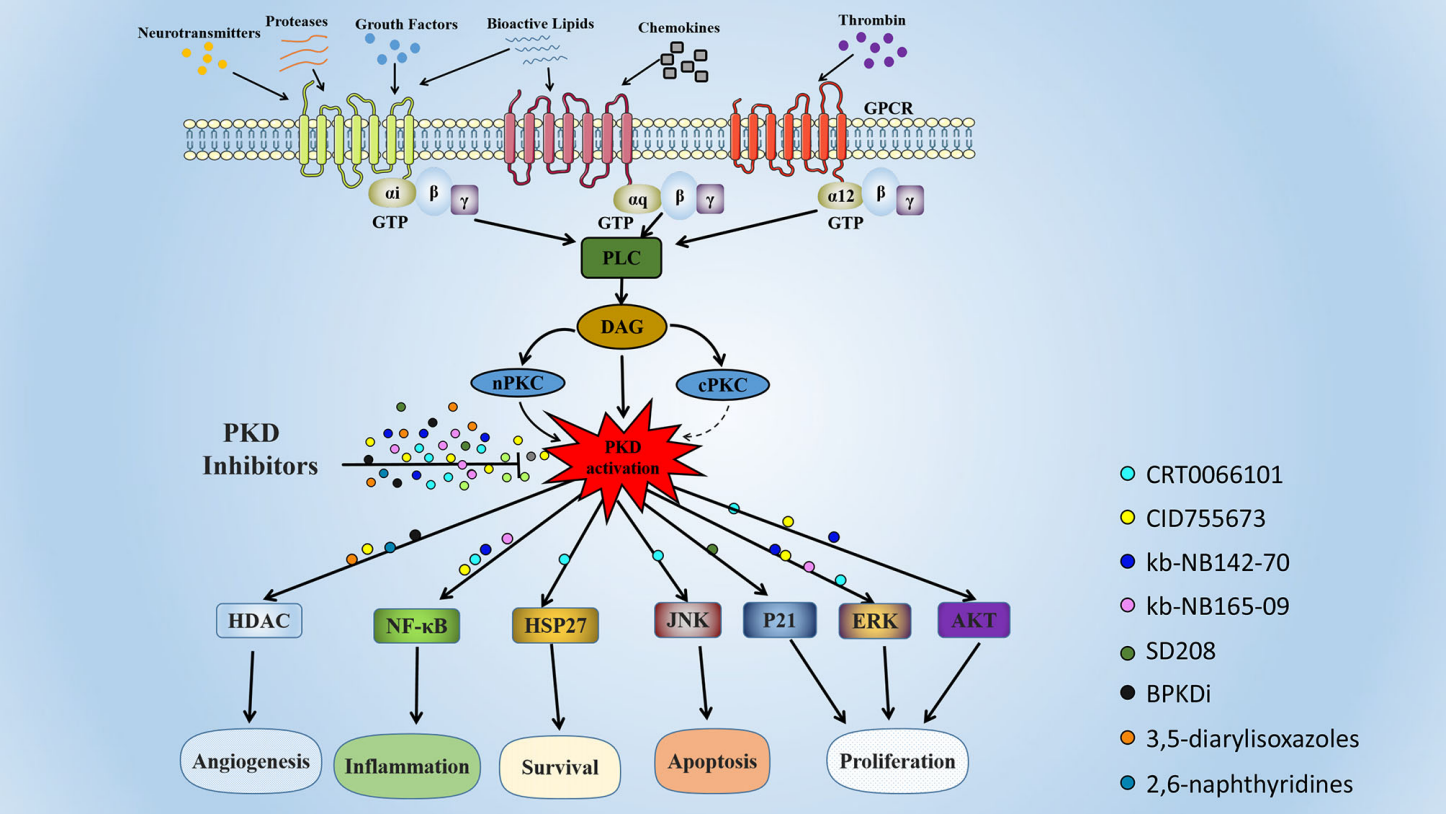
Signaling pathways and biological functions of PKD. The PKDs are activated by many substances, including phorbol ester, platelet-derived growth factor, and G protein-coupled receptor ligands. These extracellular stimuli activate intracellular phospholipase C (PLC) by binding to cell surface receptors. Activated PLC catalyzes the production of intracellular second messenger diacylglycerol (DAG), which recruits PKD and PKC to the plasma membrane. Activated PKC phosphorylates PKD at two serine residues (Ser 738 and 742 in human PKD1), resulting in PKD activation. Activated PKD regulates multiple biological functions, including angiogenesis, inflammation, cell proliferation, apoptosis, and survival. The colored globules represent the different PKD small molecule inhibitors, and the globules on the arrow represent the action of PKD inhibitors in different cellular processes.

Here, we summarized various PKD SMIs and their applications in various diseases (**Figure 3**). Although different PKD isoforms may play opposing roles in different pathological processes or even in the same disease, pan-PKD inhibitors have shown antagonistic effects *in vivo* and *in vitro* in various disease models. Various PKD inhibitors have progressed the study of various diseases, including cancer, inflammation, and obesity. These PKD specific inhibitors have shown therapeutic effects in preclinical studies and are promising targeted drug candidates.

Although PKD SMIs have shown therapeutic potential in various disease models, there are no PKD-related inhibitors currently entering clinical trials. Their use in the clinic is limited by two major factors. First, the three PKD isoforms may play different roles in diseases. There is no known comprehensive PKD crystal structure, and all PKD inhibitors to date have been pan-PKD inhibitors (19). Additional research is needed to develop PKD isoform-specific drugs that will make disease treatments more precise. Second, PKD inhibitors have off-target effects to some extent. Like most kinase inhibitors, the PKD inhibitor CRT0066101 has demonstrated activity against many other protein kinases at concentrations of 1 µM and ATP concentrations equivalent to the Km of each kinase (39). Therefore, future research must explore the crystal structure of the catalytic region of the three PKD isoforms to design SMIs with higher selectivity and fewer side effects.

## Author Contributions

Visualization, DL, YF, HC, and YK. Writing—original draft, DL, JC, and BC. Writing—review and editing, DL, PZ, ML, and JC. All authors contributed to the article and approved the submitted version.

## Funding

This work was supported by the National Natural Science Foundation of China (Grant number 81802717).

## Conflict of Interest

The authors declare that the research was conducted in the absence of any commercial or financial relationships that could be construed as a potential conflict of interest.

## Abbreviations

PKD, Protein kinase D; CaMK, Calcium/calmodulin-dependent kinase; DAG, Diacylglycerol; SMIs, Small molecule inhibitors; mTORC1-S6K1, Mammalian target of rapamycin complex 1 - S6 kinase 1; OSCC, Oral squamous cell carcinoma cells; HDAC, Histone deacetylase; HSP27, Heat shock protein 27; NF-κB, Nuclear factor kappa-light-chain-enhancer of activated B cells; VEGF, Vascular endothelial growth factor; ESCs, Embryonic stem cells; MEK, Mitogen-activated protein kinase kinase; CDKI, Cyclin-dependent kinase inhibitor; MEF_2_, Myocyte enhancer factor 2; ANF, Atrial natriuretic factor; b-MyHC, b-myosin heavy chain; a-Sk-actin, a-skeletal actin;PKC, Protein kinase C; SAR, Structure activity relationship; PDAC, Pancreatic ductal adenocarcinoma; PanINs, Pancreatic intraepithelial lesions; EGF-R, Epidermal growth factor receptor; ER, Estrogen receptor; IDC, Invasive ductal carcinomas; TNBC, Triple-negative breast cancer; CRC, Colorectal cancer; ERK, Extracellular regulated protein kinases; IL-6, Interleukin-6; MCP-1, Monocyte chemoattractant protein-1; LPA, Lysophosphatidic acid; LPAR5, LPA-receptor 5; GBS, Group B streptococcus; T2DM, Type 2 diabetes mellitus; AMPK, AMP-activated protein kinase; PLC, Phospholipase C; MMP, matrix metalloproteinase.
